# Wave trains induced by circularly polarized electric fields in cardiac tissues

**DOI:** 10.1038/srep13349

**Published:** 2015-08-25

**Authors:** Xia Feng, Xiang Gao, Juan-Mei Tang, Jun-Ting Pan, Hong Zhang

**Affiliations:** 1 Zhejiang Institute of Modern Physics and Department of Physics, Zhejiang University, Hangzhou 310027, China; 2 School of Physics and Information Technology, Shaanxi Normal University, Xi’an 710062, China; 3 Institute of Physical Oceanography and Ocean College, Zhejiang University, Hangzhou 310058, China

## Abstract

Clinically, cardiac fibrillation caused by spiral and turbulent waves can be terminated by globally resetting electric activity in cardiac tissues with a single high-voltage electric shock, but it is usually associated with severe side effects. Presently, a promising alternative uses wave emission from heterogeneities induced by a sequence of low-voltage uniform electric field pulses. Nevertheless, this method can only emit waves locally near obstacles in turbulent waves and thereby requires multiple obstacles to globally synchronize myocardium and thus to terminate fibrillation. Here we propose a new approach using wave emission from heterogeneities induced by a low-voltage circularly polarized electric field (i.e., a rotating uniform electric field). We find that, this approach can generate circular wave trains near obstacles and they propagate outwardly. We study the characteristics of such circular wave trains and further find that, the higher-frequency circular wave trains can effectively suppress spiral turbulence.

In hearts, spiral and turbulent waves may cause serious cardiac deceases, such as fibrillation^1^,^2^,^3^,^4^,^5^,^6^,^7^. At present, the clinically effective method for terminating fibrillation uses a single high-voltage electric shock to reset all electric activity in cardiac tissues^8^,^9^,^10^, but it is usually associated with severe side effects^9^,^10^,^11^. Besides this method, a theoretical effort uses local fast pacing delivered via injecting a signal on a chosen area of the heart^12^,^13^,^14^,^15^,^16^. Although this approach can numerically generate a higher-frequency wave train to suppress spiral turbulence, it is not easy to be realized in real cardiac tissues^17^,^18^.

Recently, a promising alternative called wave emission from heterogeneities (WEH) or far-field stimulation is proposed^19^,^20^,^21^. It exploits the fact that, applying an external electric field onto a whole piece of cardiac tissue can lead to de-polarizations and hyper-polarizations (so-called Weidmann zones^22^) near obstacles. These obstacles can be considered as conductivity heterogeneities inherently in cardiac tissues such as blood vessels, ischemic regions, and smaller-scale discontinuities^23^. If the de-polarizations are supra-threshold, these obstacles can act as virtual electrodes or second sources^24^,^25^,^26^,^27^,^28^,^29^,^30^. Previous works focused on WEH in response to the uniform electric field (UEF)^19^,^20^,^21^,^22^,^23^,^24^,^25^,^26^,^27^,^28^,^29^,^30^,^31^,^32^,^33^,^34^,^35^,^36^, which applies a sequence of low-voltage UEF pulses onto field electrodes. Nevertheless, WEH induced by UEF can only emit waves locally near obstacles in turbulent waves. So it requires multiple obstacles to activate more areas and progressively synchronize the whole myocardium to terminate fibrillation^19^,^20^,^21^.

Compared to UEF, the circularly polarized electric field (CPEF) has shown its unique ability to control spirals and turbulence in chemical systems^37^,^38^,^39^, which has been verified in the Belousov-Zhabotinsky reaction^40^; with a different mechanism in cardiac tissues, CPEF can also unpin the anchored spirals^41^. In this paper, we study WEH in response to CPEF, and find it can generate circular wave trains (target waves) near obstacles and they can propagate outwardly. We study the capability of CPEF to induce such circular wave trains in a quiescent medium, and analyze the angular frequency relation between the circular wave trains and CPEF. Furthermore, we present a successful application of using a higher-frequency circular wave train induced by a low-voltage CPEF to suppress spiral turbulence, and also discuss its suppression mechanism.

## Results

To describe the electric activity of cardiac tissues, we consider the following Luo-Rudy model^42^:


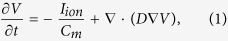



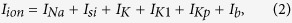


where *V* is the membrane potential, *C*_*m*_ is the membrane capacitance, *D* is the diffusion current coefficient, and *I*_*ion*_ is the total ionic currents which consist of a fast sodium current *I*_*Na*_, a slow inward current *I*_*si*_, a time-dependent potassium current *I*_*K*_, a time-independent potassium current *I*_*K*1_, a plateau potassium current *I*_*Kp*_, and a time-independent background current *I*_*b*_. In mono-domain models, the general effect of an external electric field on an obstacle can be expressed as a Neumann boundary condition^36^,^43^: **n**⋅(∇*V* − ***E***) = 0, where **n** is the normal vector to the obstacle boundary, and **E** is the external electric field. Through this paper without loss of generality, we choose **E** = (*E*_*x*_, *E*_*y*_) as a counter-clockwise rotating CPEF, where 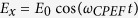, 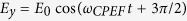, and *E*_0_, *ω*_*CPEF*_ are its strength and angular frequency, respectively.

**Figure 1:**
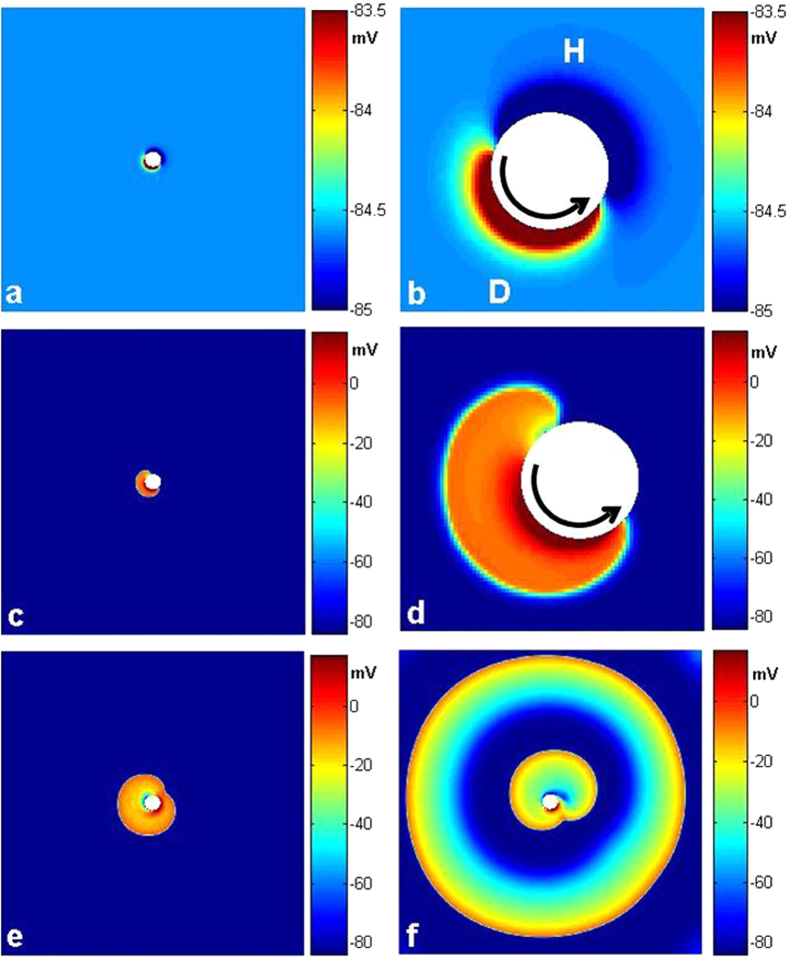
Circular waves induced by CPEF in a two-dimensional quiescent medium. The angular frequency of CPEF *ω*_*CPEF*_ = 0.14 rad/ms and the obstacle radius *R* = 0.24 cm. (**a,b**), Distribution of the membrane potential induced by CPEF with strength *E*_0_ = 0.1 V/cm. (**c,d**), A wave induced by de-polarization under CPEF with *E*_0_ = 1.0 V/cm. (**e**), The first circular wave induced by CPEF. (**f**), The second and the third circular waves induced by CPEF. The patterns in (**b**,**d**) are the enlarged views of those in (**a**,**c**), respectively. Regions **D** and **H** represent de-polarization and hyper-polarization induced by CPEF, respectively. The black curved arrows mean that CPEF rotate counter-clockwise.

In the following, we use a two-dimensional quiescent medium with a circular obstacle of radius *R* in its center to do the simulation. We find that, as shown in Fig. 1a,b, with CPEF at a weak strength *E*_0_ and certain frequency *ω*_*CPEF*_, the de-polarization and hyper-polarization induced by CPEF near the obstacle rotate synchronously with the rotating CPEF, and the membrane potential pattern is distributed similarly as Chinese “ancient Taijitu”^41^. When *E*_0_ increases above some threshold, the de-polarization begins to emit a wave as shown in Fig. 1c,d. Then the two ends of the wave propagate oppositely along the obstacle, and they quickly collide with each other and finally can form a circular wave propagating outwardly as shown in Fig. 1e. With the continued effect of CPEF, the second circular wave can be formed and it can also propagate outwardly, then the third one and so on can also emerge (see Fig. 1f). That is, a circular wave train can be generated and can continually propagate outwardly. Similarly, we also observe a circular wave train induced by CPEF near a circular obstacle in a modified FitzHugh-Nagumo model^44^, thus the above results may be model-independent. Therefore, we can recognize that an obstacle under CPEF can act as a pacing electrode and generate the circular wave train.

**Figure 2:**
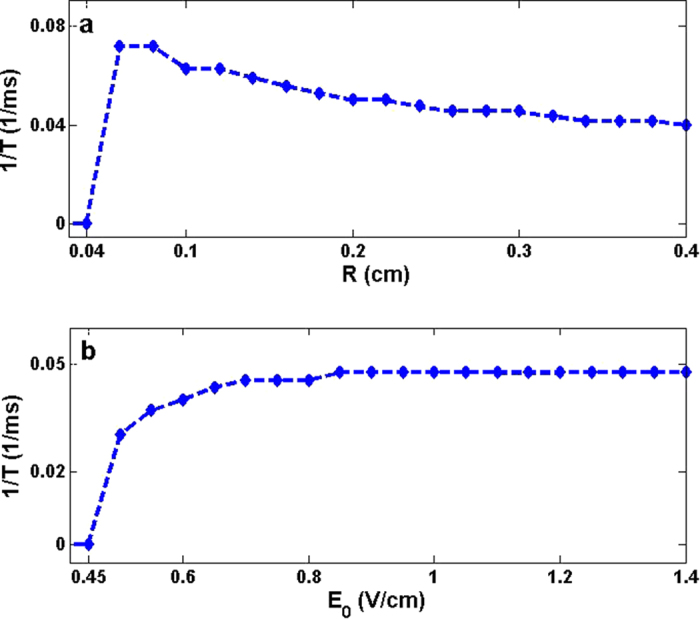
The formation time (*T*) of the first circular wave induced by CPEF in a two-dimensional quiescent medium. (**a**), The relation between 1/*T* and the obstacle radius *R*, where the strength of CPEF *E*_0_ = 1.0 V/cm. (**b**), The relation between 1/*T* and *E*_0_, where *R* = 0.24 cm. The angular frequency of CPEF *ω*_*CPEF*_ = 0.14 rad/ms in both (**a**,**b**).

As for such a circular wave train induced by CPEF, for convenience, we focus on studying the formation time (*T*) of the first circular wave to reflect the capability of CPEF to induce the circular wave train. As illustrated in Fig. 2, with a given angular frequency of CPEF, we can find *T* is highly related to *R* and *E*_0_. In details, with a given *E*_0_, the reciprocal of *T*(1/*T*) will change when *R* increases. As shown in Fig. 2a, with *E*_0_ = 1.0 V/cm, we can see *R* has a threshold at 0.04 cm, and 1/*T* has a sharp up jump. Below this threshold, 1/*T* is zero, which means no wave can be induced by CPEF near such small obstacles. Once *R* increases above this threshold, 1/*T* begins to decrease. And with the continual increasing of *R*, 1/*T* decreases more and more slowly. On the other hand, with a given *R*, 1/*T* will also change when *E*_0_ increases. As shown in Fig. 2b, with *R* = 0.24 cm, *E*_0_ also has a threshold at 0.45 V/cm, and 1/*T* also has a sharp up jump. Below this threshold, 1/*T* is zero and no wave can be induced by CPEF because the electric strength is not strong enough to exceed the de-polarization threshold. Once *E*_0_ increases above this threshold, 1/*T* begins to increase slowly. And if *E*_0_ becomes large enough (e.g. *E*_0_ = 0.85 V/cm as illustrated in Fig. 2b), 1/*T* tends to be a constant.

**Figure 3:**
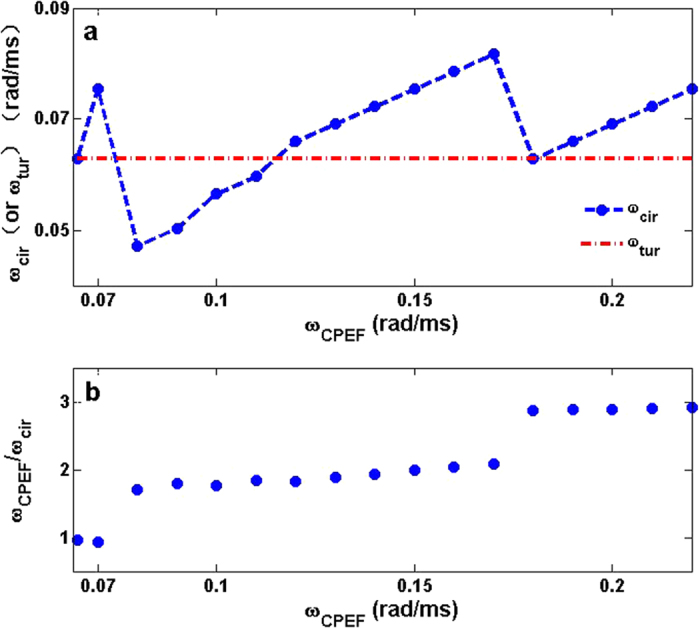
The angular frequency relations between the circular wave train and CPEF in a two-dimensional quiescent medium. (**a**), The obstacle radius *R* = 0.24 cm, the strength of CPEF *E*_0_ = 1.0 V/cm, and the angular frequency of CPEF 0.065 rad/ms ≤ *ω*_*CPEF*_ ≤ 0.22 rad/ms. The dashed line with solid circles represents the angular frequency of the circular wave trains *ω*_*cir*_. The dash-dotted line represents the dominant angular frequency of the spiral turbulence *ω*_*tur*_ in the same medium and the same obstacle. (**b**), The ratios of *ω*_*CPEF*_ over *ω*_*cir*_ correspond to the data in (**a**).

With proper *R* and *E*_0_, the circular waves can be continuously induced by CPEF and then can form a circular wave train with angular frequency *ω*_*cir*_. Since the circular wave train is forcedly excited by CPEF with *ω*_*CPEF*_, *ω*_*cir*_ should highly depend on *ω*_*CPEF*_. In Fig. 3a, to study the relation between *ω*_*cir*_ and *ω*_*CPEF*_, we gradually increase *ω*_*CPEF*_ from 0.065 rad/ms which can be seen as a minimum (Below this minimum, CPEF cannot generate a stable circular wave train). When *ω*_*CPEF*_ is set as this minimum, a stable circular wave train can be generated and its angular frequency *ω*_*cir*_ is synchronized with *ω*_*CPEF*_, and the ratio *ω*_*CPEF*_/*ω*_*cir*_ is about 1:1 as shown in Fig. 3b. However, when *ω*_*CPEF*_ increases above 0.07 rad/ms, *ω*_*cir*_ has a down jump (see Fig. 3a) which leads the ratios *ω*_*CPEF*_/*ω*_*cir*_ are no longer 1:1 but locked at nearly 2:1 (see Fig. 3b). The reason for this is that, CPEF rotates so fast that after forming a circular wave, the medium around the obstacle has not yet recovered to be excitable. Thus the rotating de-polarization of CPEF cannot excite another wave in its present round until the medium recover to be excitable again in its next round, and so forth. If *ω*_*CPEF*_ continues to increase, *ω*_*cir*_ will also increase but the ratios *ω*_*CPEF*_/*ω*_*cir*_ are always locked at nearly 2:1 until *ω*_*CPEF*_ reaches 0.17 rad/ms. When *ω*_*CPEF*_ becomes larger than 0.17 rad/ms, *ω*_*cir*_ faces another down jump and subsequently another increasing with *ω*_*CPEF*_, and the ratios *ω*_*CPEF*_/*ω*_*cir*_ are locked at nearly 3:1. In a word, as illustrated in Fig. 3, CPEF always keeps nearly *n*:1 (*n* = 1,2,3) angular frequency relation with the induced circular wave train, which has been widely reported in the pattern formation domain^45^,^46^,^47^,^48^.

Besides, we also measure the dominant angular frequency of the spiral turbulence *ω*_*tur*_ in the same medium and the same obstacle. We can see from Fig. 3a that, in some angular frequency ranges of *ω*_*CPEF*_, *ω*_*cir*_ is higher than *ω*_*tur*_. As the high-frequency waves may invade low-frequency domain^49^,^50^,^51^,^52^,^53^,^54^,^55^,^56^, we believe the circular wave trains with such higher frequencies can suppress spiral turbulence^12^,^13^,^14^,^15^,^16^,^17^,^18^.

**Figure 4:**
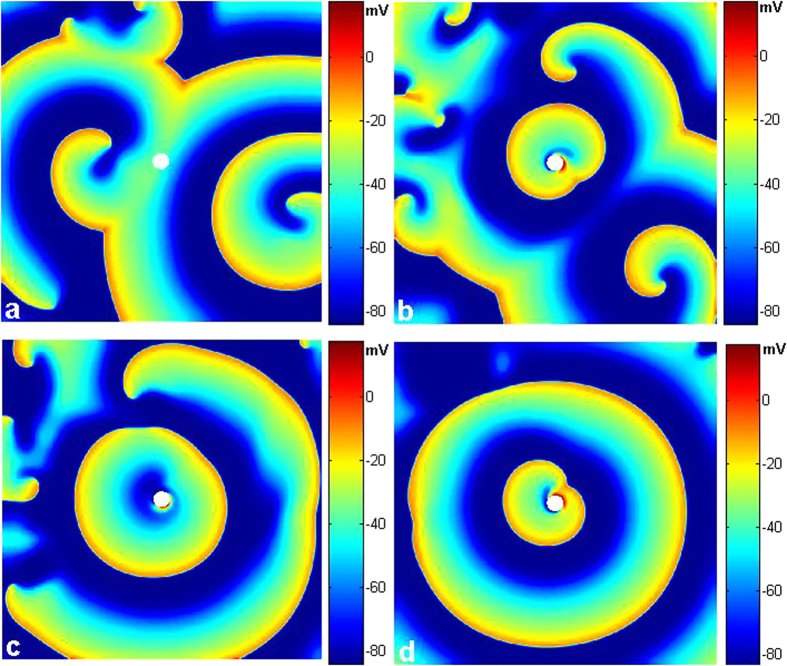
Suppression of two-dimensional spiral turbulence by CPEF. The obstacle radius *R* = 0.24 cm, and the dominant angular frequency of the spiral turbulence *ω*_*tur*_ is the same as that in Fig. 3a. The strength of CPEF *E*_0_ = 1.0 V/cm and the angular frequency of CPEF *ω*_*CPEF*_ = 0.14 rad/ms. (**a**), The CPEF is applied from *t* = 0. (**b**), *t* = 1000 m*s*. (**c**), *t* = 1800 ms. (**d**), *t* = 2800 ms.

To test this idea we choose *E*_0_ = 1.0 V/cm, *ω*_*CPEF*_ = 0.14 rad/ms for CPEF which can generate the circular wave train with angular frequency *ω*_*cir*_ higher than *ω*_*tur*_ (see Fig. 3a). In Fig. 4, we numerically simulate spiral turbulence with a circular obstacle of *R* = 0.24 cm and use it as the initial state (*t* = 0). In the beginning of applying CPEF, due to the disturbance of the nearby turbulent waves, the emitted waves near the obstacle fail to form circular waves. Nevertheless, by continuously emitting waves under CPEF, circular waves begin to emerge. Then at *t* = 1000 ms, a full circular wave is formed and squeezes out the nearby turbulent waves, following which more and more circular waves can be gradually formed. Later at *t* = 1800 ms, there are only few turbulent waves left. Finally at *t* = 2800 ms, all the turbulent waves are driven away out of the boundary. In addition, the medium can recover to a quiescent state after stopping CPEF. Furthermore, we find the circular wave trains induced by CPEF can successfully suppress spiral turbulence as long as *ω*_*cir*_ > *ω*_*tur*_. Similarly in the modified FitzHugh-Nagumo model, the higher-frequency circular wave train induced by CPEF near a circular obstacle is also obtained and it can also successfully suppress the spiral turbulence.

**Figure 5:**
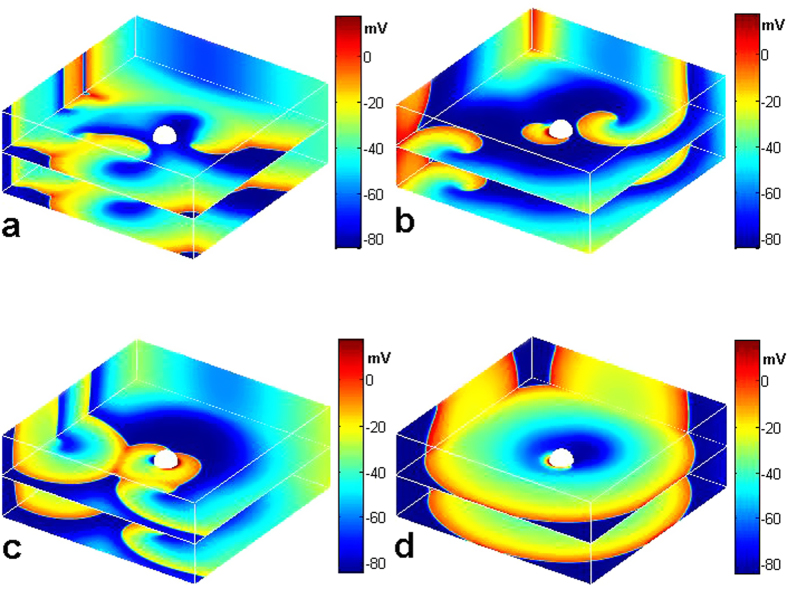
Suppression of three-dimensional scroll turbulence by CPEF. The obstacle radius *R* = 0.24 cm. The strength of CPEF *E*_0_ = 1.8 V/cm and the angular frequency of CPEF *ω*_*CPEF*_ = 0.14 rad/ms. (**a**), The CPEF is applied from *t* = 0. (**b**), *t* = 650 m*s*. (**c**), *t* = 890 ms. (**d**), *t* = 1100 ms.

Furthermore, we also testify the ability of CPEF suppressing three-dimensional scroll turbulence in Luo-Rudy model. As illustrated in Fig. 5, we numerically simulate scroll turbulence with a spherical obstacle of *R* = 0.24 cm as the initial state (*t* = 0). Then under CPEF, emitting waves continuously emerge and collide with the turbulent waves (*t* = 650 ms). Later at *t* = 890 ms, a full spherical wave is formed and squeezes out the nearby turbulent waves. Finally at *t* = 1100 ms, all the turbulent waves are driven away out of the boundary. Similarly, we also observe that, in the modified FitzHugh-Nagumo model, a spherical wave train induced by CPEF near a spherical obstacle can successfully suppress the three-dimensional scroll turbulence.

## Discussion

**Figure 6:**
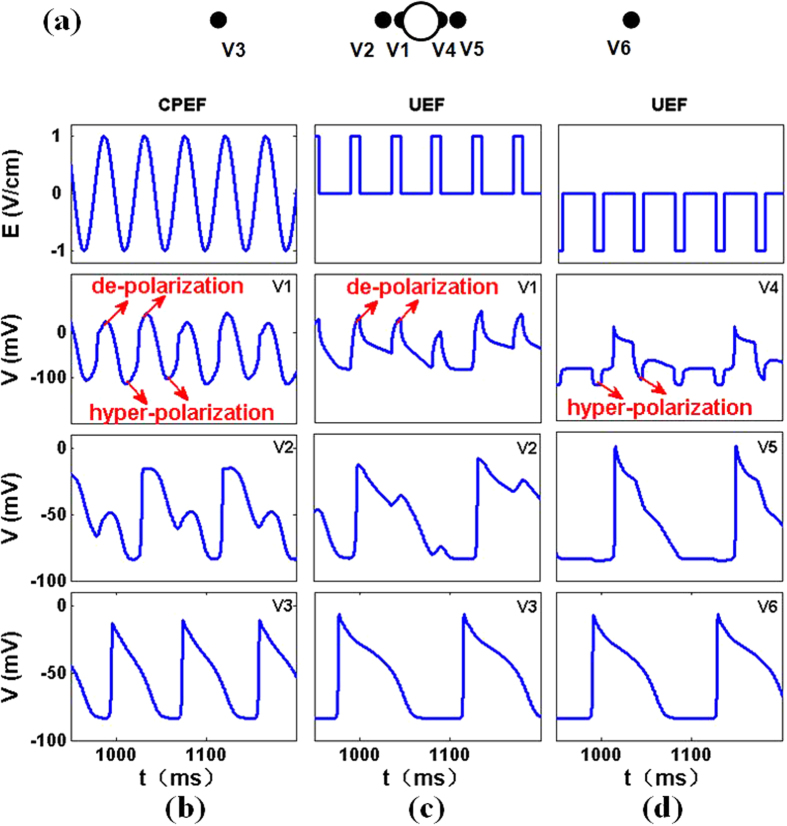
The variations of the membrane potentials V under CPEF or UEF in a two-dimension quiescent medium. (**a**), The locations of the membrane potentials V1-V6. V1, V4 are the membrane potentials on the obstacle boundary. V2, V5 are the membrane potentials near the obstacle boundary. V3, V6 are the membrane potentials far away from the obstacle boundary. CPEF rotates counter-clockwise and UEF is horizontal. (**b**), Under CPEF, the strength *E*_0_ = 1.0 V/cm and the angular frequency *ω*_*CPEF*_ = 0.14 rad/ms. (**c,d),** Under UEF, *E*_0_ = 1.0 V/cm, *ω*_*UEF*_ = 0.14 rad/ms, and the pluse duration is 10 ms. The red arrows indicate the effects of the de-polarizations and hyper-polarizations.

In this section, we discuss the mechanism about successfully suppressing the spiral turbulence by a high-frequency circular wave train induced by CPEF. And we can owe this success to the contributions of the rotating de-polarization and hyper-polarization induced by CPEF, thus the membrane potential at the boundary of the obstacle would successively and periodically go through both effects. Under the influence of CPEF (*E*_0_ = 1.0 V/cm, *ω*_*CPEF*_ = 0.14 rad/ms, the same as those in Fig. 4), we study the variation of the membrane potential at an arbitrary position on the obstacle boundary in a quiescent medium, e.g., the membrane potential V1 in Fig. 6a. We find V1 would be depolarized to the excited state, and then forced to recover to the excitable state quickly by the hyper-polarization as shown in Fig. 6b. Due to the diffusion of V1, the nearby membrane potential V2 would also be directly affected. Although the second stimulus from V1 fails to de-polarize V2, the effect of hyper-polarization from V1 still makes it recovered to the excitable state quickly. Thus V2 is able to be de-polarized by the third stimulus from V1. Further, the membrane potential V3 would be affected by the diffusion of V2 and thus forms periodic excitations (i.e., the circular wave train). Hence every two rounds of the rotating de-polarization and hyper-polarization induced by CPEF can stimulate a circular wave. The angular frequency of the circular wave train *ω*_*cir*_ is 0.072 rad/ms, which is higher than *ω*_*tur*_. Therefore, the circular wave train induced by CPEF with such a high angular frequency can be used to suppress the spiral turbulence.

As shown in Fig. 1A of Ref. 35, using UEF can also generate circular waves near the obstacle in a quiescent medium. However, UEF can hardly utilize the same mechanism as CPEF to induce a higher-frequency circular wave train for suppressing the spiral turbulence. We employ a series of UEF pulses to the quiescent medium with the same electric strength and angular frequency as CPEF. In Fig. 6c, we find the membrane potential V1 excited by the de-polarization induced by UEF cannot be forced to recover to the excitable state quickly due to the lack of the hyper-polarization in the same position, thus the nearby membrane potential V2 will have to go through a relatively long excited time. Hence the membrane potential V2 and thereby V3 can only be stimulated for every three UEF pulses. As illustrated in Fig. 6d, the membrane potential V4 is only affected by the hyper-polarization induced by UEF which cannot induce stimuli and the existing stimuli actually come from V1. So the ratio *ω*_*UEF*_/*ω*_*cir*_ is about 3:1 and *ω*_*cir*_ is about 0.047 rad/ms, which is lower than *ω*_*tur*_. Therefore UEF at *ω*_*UEF*_ = 0.14 rad/ms cannot induce a higher-frequency circular wave train to suppress the spiral turbulence.

**Figure 7:**
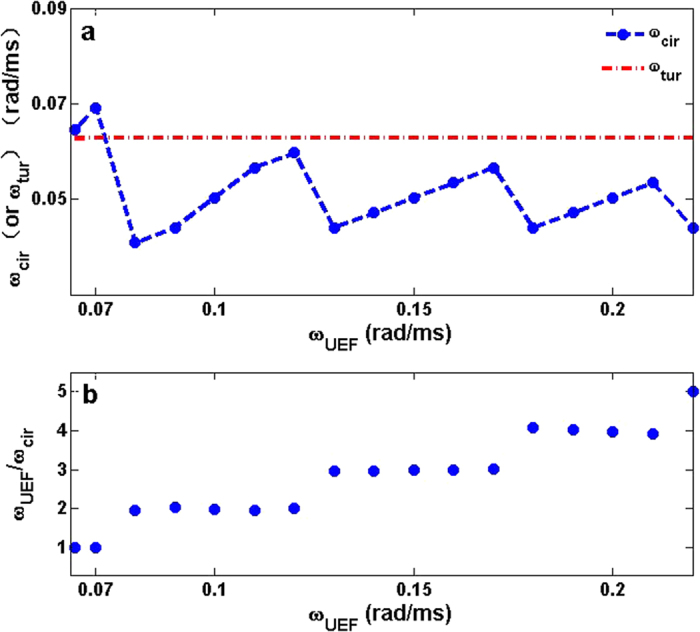
The angular frequency relations between the circular wave trains and UEF in a two-dimension quiescent medium. (**a**), The obstacle radius *R* = 0.24 cm, the strength of UEF *E*_0_ = 1.0 V/cm, the angular frequency of UEF 0.065 rad/ms ≤ *ω*_*UEF*_ ≤ 0.22 rad/ms, and the pluse duration is 10 ms. The dashed line with solid circles represents the angular frequency of the circular wave trains *ω*_*cir*_. The dash-dotted line represents the dominant angular frequency of the spiral turbulence *ω*_*tur*_ in the same medium and the same obstacle. (**b**), The ratios of *ω*_*UEF*_ over *ω*_*cir*_ correspond to the data in (**a**).

In order to verify whether UEF with other *ω*_*UEF*_ can induce higher-frequency circular wave trains, we measure *ω*_*cir*_ in a large region of *ω*_*UEF*_ in the same quiescent medium and the same obstacle as in Fig. 3a. As shown in Fig. 7a, most of the circular wave trains induced by UEF have *ω*_*cir*_ < *ω*_*tur*_ and thus cannot suppress the spiral turbulence. Comparing it to the case of CPEF in Fig. 3a and taking the angular frequency ranges of 0.13 rad/ms ≤ *ω* ≤ 0.17 rad/ms for instance, we find every two rounds of CPEF can stimulate a circular wave, but UEF at the same angular frequency would need three pulses to stimulate a circular wave (may refer to Figs 3b and 7b), and thus *ω*_*cir*_(*UEF*) < *ω*_*tur*_ < *ω*_*cir*_(*CPEF*).

In other words, the main difference of CPEF from UEF is the rotation. Because of the rotation of CPEF, the medium will be affected by both de-polarization and hyper-polarization. While using UEF, only the de-polarization can affect the medium. Therefore, with the same angular frequency of both external electric fields (*ω* = 0.14 rad/ms in Fig. 6), the ratio *ω*_*CPEF/*_*ω*_cir_ is about 2:1, as shown in Fig. 6b. However, the ratio *ω*_*UEF/*_*ω*_cir_ is about 3:1, as illustrated in Fig. 6c. Hence the circular wave train induced by the rotating CPEF has the angular frequency of *ω*_*cir*_(CPEF) = 0.072 rad/ms which is higher than *ω*_*cir*_(UEF) = 0.047 rad/ms. And this difference of ratios between CPEF and UEF exists in a wide region of the angular frequency *ω* of both external electric fields. Comparing with the Figs 3a and 7a, the rotating CPEF can maintain the phase-locking state at the ratio of 2:1 in a longer region than UEF. And in some part of the ratio of 2:1 (i.e., 0.13 rad/ms ≤*ω* ≤ 0.17 rad/ms), the circular wave trains induced by CPEF have higher frequencies than the dominant frequency of turbulence and can be used to terminate fibrillation. But in the same region (i.e.,0.13 rad/ms ≤ *ω* ≤0.17 rad/ms), the circular wave trains induced by UEF are at the ratio of 3:1, and thus have lower frequencies than the dominant frequency of turbulence, and cannot be used to terminate fibrillation.

Moreover, further simulations indicate the waves induced by UEF cannot form the circular waves in the turbulent waves and thereby the induced circular wave trains in the quiescent medium with relatively high angular frequencies (*ω*_*cir*_ = 0.07 rad/ms in Fig. 7) also cannot suppress the spiral turbulence as in Fig. 4. This may owe to the fact that the de-polarization and hyper-polarization induced by UEF cannot rotate and thus the waves can only be induced in a fixed position near the obstacle (e.g., only V1 in Fig. 6c affected by the de-polarization can emit waves while V4 in Fig. 6d cannot). Conversely, the de-polarization and hyper-polarization induced by CPEF can rotate and thereby the waves can be emitted in any position on the obstacle boundary (e.g., the membrane potential at the arbitrary position on the obstacle boundary has the same variation as V1 in Fig. 6b). Hence CPEF can effectively generate the circular wave trains and eventually suppress the turbulent waves. Therefore, although the circular wave trains can be induced by UEF in a quiescent medium, they cannot form the circular waves in the presence of turbulence waves as in Fig. 4 and thereby UEF can hardly utilize the same mechanism as CPEF to suppress spiral turbulence.

To conclude, CPEF can effectively generate the higher-frequency circular wave trains near obstacles. And this capability is closely related to the strength and the angular frequency of CPEF and the size of obstacles. Moreover, the circular wave trains induced by CPEF have a wide application prospect. An important application is that the higher-frequency circular wave trains induced by CPEF can be used to suppress spiral turbulence, which may provide a promising alternative to terminate fibrillation. Additionally, CPEF has been realized in Belousov-Zhabotinsky reaction by applying two ACs onto two pairs of field electrodes perpendicular to each other^40^. Similarly, it will also be easily realized in cardiac tissues by replacing DCs to ACs in the experimental preparation of Fig. 5D in Ref. 20. Hence we believe this approach will have strong practical value in heart clinical treatments, and its effectiveness and applicability in bi-domain model and in real cardiac tissues will need to be further studied.

## Methods

In Luo-Rudy model, to add the introduced boundary condition into the circular boundary of the obstacle in Cartesian coordinates, we adopt the phase field method^36^,^43^. Considering the effect of an external electric field on the obstacle, equation (1) can be adapted as


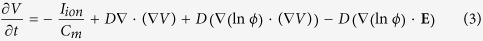


where *C*_*m*_ = 1 μF/cm^2^, *D* = 0.001 cm^2^/ms, the total ionic currents *I*_*ion*_ are determined by ionic gates, whose gating variables are obtained as solutions to a coupled system of nonlinear ordinary differential equations, and the parameters are modified as in Ref. 57. In Cartesian coordinates, equation (3) is integrated on the 10 cm × 10 cm two-dimensional medium and 5 cm × 5 cm × 2 cm three-dimensional medium which are large enough to sustain the turbulence^57^,^58^,^59^ with no-flux boundary conditions via Euler method, and the central difference method is applied to compute the Laplacian term ∇^2^*V* and the gradient terms 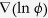, ∇*V*. The space and the time step in two-dimensional domain are Δ*x* = 0.015 cm, Δ*y* = 0.015 cm and Δ*t* = 0.005 ms, respectively. And the space and the time step in three-dimensional domain are Δ*x* = 0.02 cm, Δ*y* = 0.02 cm, Δ*z* = 0.02 cm and Δ*t* = 0.01 ms, respectively.

## Additional Information

**How to cite this article**: Feng, X. *et al.* Wave trains induced by circularly polarized electric fields in cardiac tissues. *Sci. Rep.* **5**, 13349; doi: 10.1038/srep13349 (2015).

## References

[b1] Winfree A. T. When Time Breaks Down. (Princeton NJ: Princeton University Press, 1987).

[b2] Davidenko J. M., Pertsov A. V., Salomonsz R., Baxter W. & Jalife J. Stationary and drifting spiral waves of excitation in isolated cardiac-muscle. Nature 355, 349–351 (1992). 1731248 10.1038/355349a0

[b3] Gray R. A., Pertsov A. M. & Jalife J. Spatial and temporal organization during cardiac fibrillation. Nature 392, 75–78 (1998). 9510249 10.1038/32164

[b4] Witkowski F. X. *et al.* Spatiotemporal evolution of ventricular fibrillation. Nature 392, 78–82 (1998). 9510250 10.1038/32170

[b5] Jalife J. Ventricular fibrillation: mechanisms of initiation and maintenance. Annu. Rev. Physiol. 62, 25–50 (2000). 10845083 10.1146/annurev.physiol.62.1.25

[b6] Cherry E. M. & Fenton F. H. Visualization of spiral and scroll waves in simulated and experimental cardiac tissue. New J. Phys. 10, 125016 (2008).

[b7] Karma A. Physics of cardiac arrhythmogenesis. Annu. Rev. Condens. Matter Phys. 4, 313–337 (2013).

[b8] Koster R. W. *et al.* A randomized trial comparing monophasic and biphasic waveform shocks for external cardioversion of atrial fibrillation. Am. Heart J. 147, e1–e7 (2004). 15131555 10.1016/j.ahj.2003.10.049

[b9] Babbs C. F., Tacker W. A., VanVleet J. F., Bourland J. D. & Geddes L. A. Therapeutic indices for transchest defibrillator shocks: effective, damaging, and lethal electrical doses. Am. Heart J. 99, 734–738 (1980). 7377095 10.1016/0002-8703(80)90623-7

[b10] Santini M. *et al.* Single shock endocavitary lowenergy intracardiac cardioversion of chronic atrial fibrillation. J. Interv. Card. Electrophysiol. 3, 45–51 (1999). 10354975 10.1023/a:1009871422517

[b11] Walcott G. P., Killingsworth C. R. & Ideker R. E. Do clinically relevant transthoracic defibrillation energies cause myocardial damage and dysfunction? Resuscitation 59, 59–70 (2003). 14580735 10.1016/s0300-9572(03)00161-8

[b12] Zhang H., Hu B. B. & Hu G. Suppression of spiral waves and spatiotemporal chaos by generating target waves in excitable media. Phys. Rev. E 68, 026134 (2003). 10.1103/PhysRevE.68.026134 14525076

[b13] Zhang H., Cao Z. J., Wu N. J., Ying H. P. & Hu G. Suppress Winfree turbulence by local forcing excitable systems. Phys. Rev. Lett. 94, 188301 (2005). 15904413 10.1103/PhysRevLett.94.188301

[b14] Yuan G. Y., Wang G. R. & Chen S. G. Control of spiral waves and spatiotemporal chaos by periodic perturbation near the boundary. Europhys. Lett. 72, 908–914 (2005).

[b15] Cao Z. J., Li P. F., Zhang H., Xie F. G. & Hu G. Turbulence control with local pacing and its implication in cardiac defibrillation. Chaos 17, 015107 (2007). 17411264 10.1063/1.2713688

[b16] Tang G. N., Deng M. Y., Hu B. B. & Hu G. Active and passive control of spiral turbulence in excitable media. Phys. Rev. E 77, 046217 (2008). 10.1103/PhysRevE.77.046217 18517720

[b17] Wathen M. S. *et al.* Prospective randomized multicenter trial of empirical antitachycardia pacing versus shocks for spontaneous rapid ventricular tachycardia in patients with implantable cardioverter-defibrillators—Pacing Fast Ventricular Tachycardia Reduces Shock Therapies (PainFREE Rx II) trial results. Circulation 110, 2591–2596 (2004). 15492306 10.1161/01.CIR.0000145610.64014.E4

[b18] Qu Z. L., Hu G., Garfinkel A. & Weiss J. N. Nonlinear and stochastic dynamics in the heart, Phys. Rep. 543, 61–162 (2014). 25267872 10.1016/j.physrep.2014.05.002 PMC4175480

[b19] Pumir A. *et al.* Wave emission from heterogeneities opens a way to controlling chaos in the heart. Phys. Rev. Lett. 99, 208101 (2007). 18233188 10.1103/PhysRevLett.99.208101

[b20] Fenton F. H. *et al.* Termination of atrial fibrillation using pulsed low-energy far-field stimulation. Circulation 120, 467–476 (2009). 19635972 10.1161/CIRCULATIONAHA.108.825091 PMC2867100

[b21] Luther S. *et al.* Low-energy control of electrical turbulence in the heart. Nature 475, 235–239 (2011). 21753855 10.1038/nature10216 PMC3153959

[b22] Weidmann S. Effect of current flow on the membrane potential of cardiac muscle. J. Physiol. 115, 227–236 (1951). 14898488 10.1113/jphysiol.1951.sp004667 PMC1391998

[b23] Plonsey R. The nature of sources of bioelectric and biomagnetic fields. Biophys. J. 39, 309–312 (1982). 7139030 10.1016/S0006-3495(82)84521-9 PMC1328948

[b24] Sepulveda N. G., Roth B. J. & Wikswo J. P. Current injection into a two-dimensional anisotropic bidomain. Biophys. J. 55, 987–999 (1989). 2720084 10.1016/S0006-3495(89)82897-8 PMC1330535

[b25] Sobie E. A., Susil R. C. & Tung L. A generalized activating function for predicting virtual electrodes in cardiac tissue. Biophys. J. 73, 1410–1423 (1997). 9284308 10.1016/S0006-3495(97)78173-6 PMC1181040

[b26] Fishler M. G. Syncytial heterogeneity as a mechanism underlying cardiac far-field stimulation during defibrillation-level shocks. J. Cardiovasc. Electr. 9, 384–394 (1998). 10.1111/j.1540-8167.1998.tb00926.x 9581954

[b27] Fast V. G., Rohr S., Gillis A. M. & Kléber A. G. Activation of cardiac tissue by extracellular electrical shocks: Formation of ‘secondary sources’ at intercellular clefts in monolayers of cultured myocytes. Circ. Res. 82, 375–385 (1998). 9486666 10.1161/01.res.82.3.375

[b28] Trayanova N. & Skouibine K. Modeling defibrillation—Effects of fiber curvature. J. Electrocardiol. 31, 23–29 (1998). 9988001 10.1016/s0022-0736(98)90274-6

[b29] Hooks D. A. *et al.* Cardiac microstructure: Implications for electrical propagation and defibrillation in the heart. Circ. Res. 91, 331–338 (2002). 12193466 10.1161/01.res.0000031957.70034.89

[b30] Woods M. C. *et al.* Virtual electrode effects around an artificial heterogeneity during field stimulation of cardiac tissue. Heart Rhythm 3, 751–752 (2006). 16731485 10.1016/j.hrthm.2005.11.003

[b31] Pumir A. & Krinsky V. Unpinning of a rotating wave in cardiac muscle by an electric field. J. Theor. Biol. 199, 311–319 (1999). 10433895 10.1006/jtbi.1999.0957

[b32] Takagi S. *et al.* Unpinning and removal of a rotating wave in cardiac muscle. Phys. Rev. Lett. 93, 058101 (2004). 15323732 10.1103/PhysRevLett.93.058101

[b33] Ripplinger C. M., Krinsky V. I., Nikolski V. P. & Efimov I. R. Mechanisms of unpinning and termination of ventricular tachycardia. Am. J. Physiol. Heart Circ. Physiol. 291, H184–H192 (2006). 16501014 10.1152/ajpheart.01300.2005

[b34] Bittihn P. *et al.* Far field pacing supersedes anti-tachycardia pacing in a generic model of excitable media. New J. Phys. 10, 103012 (2008).

[b35] Cysyk J. & Tung L. Electric field perturbations of spiral waves attached to millimeter-size obstacles. Biophys. J. 94, 1533–1541 (2008). 17921205 10.1529/biophysj.107.116244 PMC2212699

[b36] Bittihn P., Hörning M. & Luther S. Negative curvature boundaries as wave emitting sites for the control of biological excitable media. Phys. Rev. Lett. 109, 118106 (2012). 23005683 10.1103/PhysRevLett.109.118106

[b37] Cai M. C., Pan J. T. & Zhang H. Electric-field-sustained spiral waves in subexcitable media. Phys. Rev. E 86, 016208 (2012). 10.1103/PhysRevE.86.016208 23005508

[b38] Li B. W., Deng L. Y. & Zhang H. Chiral symmetry breaking in a reaction-diffusion system. Phys. Rev. E 87, 042905 (2013). 10.1103/PhysRevE.87.042905 23679487

[b39] Li B. W., Cai M. C., Zhang H., Panfilov A. V. & Dierckx H. Chiral selection and frequency response of spiral waves in reaction-diffusion systems under a chiral electric field. J. Chem. Phys. 140, 184901 (2014). 24832300 10.1063/1.4874645

[b40] Ji L., Zhou Y., Li Q., Qiao C. & Ouyang Q. Experimental evidence of using a circularly polarized electric field to control spiral turbulence. Phys. Rev. E 88, 042919 (2013). 10.1103/PhysRevE.88.042919 24229261

[b41] Feng X., Gao X., Pan D. B., Li B. W. & Zhang H. Unpinning of rotating spiral waves in cardiac tissues by circularly polarized electric fields. Sci. Rep. 4, 04831 (2014). 10.1038/srep04831 PMC4003477 24777360

[b42] Luo C. H. & Rudy Y. A model of the ventricular cardiac action potential depolarization, repolarization, and their interaction. Circ. Res. 68, 1501–1526 (1991). 1709839 10.1161/01.res.68.6.1501

[b43] Bittihn P. Complex structure and dynamics of the heart, PhD thesis, Georg-August-Universität Göttingen (2013).

[b44] Bär M. & Eiswirth M. Turbulence due to spiral breakup in a continuous excitable medium. Phys. Rev. E 48, R1635–R1637 (1993). 10.1103/physreve.48.r1635 9960866

[b45] Petrov V., Ouyang Q. & Swinney H. L. Resonant pattern formation in a chemical system. Nature 388, 655 (1997).

[b46] Lin A. L., Bertram M., Martinez K. & Swinney H. L. Resonant phase patterns in a reaction-diffusion system. Phys. Rev. Lett. 84, 4240 (2000). 10990655 10.1103/PhysRevLett.84.4240

[b47] Steinbock O., Zykov V. & Müller S. C. Control of spiral-wave dynamics in active media by periodic modulation of excitability. Nature 366, 322 (1993).

[b48] Braune M., Schrader A. & Engel H. Entrainment and resonance of spiral waves in active media with periodically modulated excitability. Chem. Phys. Lett. 222, 358 (1994).

[b49] Hendrey M., Ott E. & Antonsen T. M. Jr. Effect of inhomogeneity on spiral wave dynamics. Phys. Rev. Lett. 82, 859 (1999).

[b50] Hendrey M., Ott E. & Antonsen T. M. Jr. Spiral wave dynamics in oscillatory inhomogeneous media. Phys. Rev. E 61, 4943 (2000). 10.1103/physreve.61.4943 11031537

[b51] Lee K. J. Wave pattern selection in an excitable system. Phys. Rev. Lett. 79, 2907 (1997).

[b52] Xie F. G., Qu Z. L., Weiss J. N. & Garfinkel A. Interactions between stable spiral waves with different frequencies in cardiac tissue. Phys. Rev. E 59, 2203 (1999).

[b53] Zhang C. X., Liao H. M., Zhou L. Q. & Ouyang Q. Pattern selection in the Belousov-Zhabotinsky reaction with the addition of an activating reactant. J. Phys. Chem. B 108, 16990 (2004).

[b54] Smolka L. B., Marts B. & Lin A. L. Effect of inhomogeneities on spiral wave dynamics in the Belousov-Zhabotinsky reaction. Phys. Rev. E 72, 056205 (2005). 10.1103/PhysRevE.72.056205 16383725

[b55] Kheowan O.-U., Mihaliuk E., Blasius B., Sendiña-Nadal I. & Showalter K. Wave mediated synchronization of nonuniform oscillatory media. Phys. Rev. Lett. 98, 074101 (2007). 17359024 10.1103/PhysRevLett.98.074101

[b56] Luo J. M., Zhang B. S. & Zhan M. Frozen state of spiral waves in excitable media. Chaos 19, 033133 (2009). 19792013 10.1063/1.3224034

[b57] Qu Z. L., Xie F. G., Garfinkel A. & Weiss J. N. Origins of spiral wave meander and breakup in a two-dimensional cardiac tissue model. Ann. Biomed. Eng. 28, 755–771 (2000). 11016413 10.1114/1.1289474

[b58] Qu Z. L., Kil J., Xie F. G., Garfinkel A. & Weiss J. N. Scroll wave dynamics in a three-dimensional cardiac tissue model: roles of restitution, thickness, and fiber rotation. Biophys. J. 78, 2761–2775 (2000). 10827961 10.1016/S0006-3495(00)76821-4 PMC1300866

[b59] Alonso S. & Panfilov A. V. Negative filament tension in the Luo-Rudy model of cardiac tissue. Chaos 17, 015102 (2007). 17411259 10.1063/1.2430638

